# Familial Mediterranean fever is no longer a rare disease in Japan

**DOI:** 10.1186/s13075-016-1071-5

**Published:** 2016-07-30

**Authors:** Kiyoshi Migita, Yasumori Izumi, Yuka Jiuchi, Nozomi Iwanaga, Chieko Kawahara, Kazunaga Agematsu, Akihiro Yachie, Junya Masumoto, Keita Fujikawa, Satoshi Yamasaki, Tadashi Nakamura, Yoshifumi Ubara, Tomohiro Koga, Yoshikazu Nakashima, Toshimasa Shimizu, Masataka Umeda, Fumiaki Nonaka, Michio Yasunami, Katsumi Eguchi, Koh-ichiro Yoshiura, Atsushi Kawakami

**Affiliations:** 1 Department of Rheumatology, Fukushima Medical University School of Medicine, Hikarigaoka 1, Fukushima, Fukushima 960-1295 Japan; 2 Clinical Research Center, NHO Nagasaki Medical Center, Kubara 2-1001-1, Omura, Nagasaki 856-8562 Japan; 3 Department of Infectious Immunology, Shinshu University Graduate School of Medicine, Asahi 3-1-1, Matsumoto, Nagano 390-8621 Japan; 4 Department of Pediatrics, School of Medicine, Institute of Medical, Pharmaceutical and Health Sciences, Kanazawa University, Takara13-1, Kanazawa, 920-8640 Japan; 5 Department of Pathology, Ehime University Proteo-Science Center and Graduate School of Medicine, Shitsukawa 454, Toon, Ehime 791-0295 Japan; 6 Department of Rheumatology, Japan Community Health care Organization, Isahaya General Hospital, Eishohigashi-machi 24-1, Isahaya, 854-8501 Japan; 7 Department of Rheumatology, Hiroshima University Hospital, Kasumi 1-2-3, Minami-ku, Hiroshima 734-8551 Japan; 8 Department of Rheumatology, Kumamoto Shinto General Hospital, Shinyashiki 1-17-27, Kumamoto, 862-8655 Japan; 9 Department of Rheumatology, Toranomon Hospital, Toranomon 2-2-2, Minato-ku, Tokyo 105-8470 Japan; 10 Department of Immunology and Rheumatology, Unit of Translational Medicine, Graduate School of Biomedical Sciences, Nagasaki University, Sakamoto1-7-1, Nagasaki, 852-8501 Japan; 11 Departments of Rheumatology, Sasebo City General Hospital, Hirase 9-3, Sasebo, 857-8511 Japan; 12 Department of Clinical Medicine, Institute of Tropical Medicine, Nagasaki University, Sakamoto 1-7-1, Nagasaki, 852-8501 Japan; 13 Sasebo Chuo Hospital, Yamato 15, Sasebo, 857-1195 Japan; 14 Department of Human Genetics, Atomic Bomb Disease Institute, Nagasaki University, Sakamoto 1-12-4, Nagasaki, 852-8523 Japan

**Keywords:** Familial Mediterranean fever, *MEFV* gene, Rheumatic manifestations

## Abstract

**Background:**

The aim of this study was to evaluate the clinical manifestations and prevalence of familial Mediterranean fever (FMF) in Japanese patients with unexplained fever and rheumatic manifestations.

**Methods:**

We enrolled 601 patients with unexplained fever or suspected FMF throughout Japan between 2009 and 2015. Patients were divided into three groups according to Tel Hashomer criteria: sure FMF, probable FMF, and non-FMF patients, including definitive rheumatic diseases. Mutation detection in exons 1, 2, 3, and 10 of the FMF gene *MEFV* was performed by direct sequencing.

**Results:**

A total of 192 patients (31.9 %) were diagnosed with FMF according to FMF diagnostic criteria. These could be divided into sure FMF (56.3 %, *n* = 108) and probable FMF (43.7 %, *n* = 84) patients. Fever, abdominal symptoms, and thoracic symptoms were significantly more common in FMF than non-FMF patients. Among FMF patients, 26 (13.5 %) had concomitant rheumatic diseases. Most FMF patients (94.3 %, 181/192) carried at least one *MEFV* mutation. Allele frequencies of M694I (13.5 % vs 0 %) and E148Q (39.1 % vs 24.8 %) mutations were significantly higher in FMF compared with healthy subjects. Allele frequencies of common *MEFV* mutations in FMF patients were M694I (13.5 %), P369S (8.6 %), R408Q (8.1 %), G304R (2.9 %), R202Q (4.4 %), E148Q (39.1 %), L110P (11.7 %), and E84K (3.1 %). Patients with a sure FMF phenotype had a higher frequency of *MEFV* exon 10 mutation (M694I) and a lower frequency of *MEFV* exon 3 mutations (P369S, R408Q) compared with those with a probable FMF phenotype.

**Conclusion:**

The high prevalence of FMF in Japanese patients with unexplained fever was confirmed in the present study. FMF should be suspected in cases of unexplained fever or non-specific rheumatic manifestations, and mutational analysis of *MEFV* could be useful to predict the clinical phenotypes of FMF in Japan.

## Background

Familial Mediterranean fever (FMF) is an autosomal recessive disorder characterized by short, recurrent bouts of fever [1]. The recurrent episodes of fever and systemic inflammation, which last a few days and commonly appear during pre-adolescence, are accompanied by peritonitis, arthritis, pleurisy, and skin manifestations [2]. FMF diagnosis is difficult because of the lack of specific clinical signs. It is prevalent in Mediterranean and Middle Eastern populations [3], where clinical diagnosis has been prompt, but non-Mediterranean FMF patients have also been reported [4]. Although considered a rare disease, it is possible that its diagnosis has been delayed in some countries such as Japan [5].

Molecular genetic diagnostic testing is often used to provide some information on FMF diagnosis [6]. However, a crucial issue for genetic counseling is that some patients presenting with manifestations of sure FMF are heterozygotes of *MEFV* variants [7]. The identification of double *MEFV* mutations in patients with FMF symptoms confirms the disease analysis, but it is not uncommon for no mutated alleles or only a single mutated allele to be detected, even in Mediterranean FMF patients [8]. Moreover, in Japanese FMF patients, *MEFV* exon 10 mutations are usually associated with sure disease phenotypes, even in heterozygous carriage [9]. A high proportion of asymptomatic carriers of *MEFV* exon 2 or 3 variants is also observed [10, 11].

This observational study was performed to determine the actual prevalence of FMF in Japanese patients with unexplained fever and to elucidate its clinical characteristics. We also analyzed the implications of these *MEFV* variants on the clinical picture of Japanese patients with unexplained fever or non-specific rheumatic manifestations.

## Methods

### Design, setting, patients, and measurements

The study was conducted at the Clinical Research Center of Nagasaki Medical Center, Japan. Patients with unexplained fever were recruited consecutively from those treated and followed up in the rheumatology department of participating hospitals. Unexplained fever was defined as a temperature above 38 °C that lasts for 3 weeks including recurrent episodes of fever without diagnosis after standardized history-taking, physical examination, and obligatory investigation. These subjects included the newly diagnosed FMF patients in the previously performed multi-centric survey for FMF [12]. The study comprised 601 patients (216 males, 385 females, mean age 44.3 ± 20.2 years). On the basis of Tel Hashomer criteria [13], patients were divided into three groups: sure FMF—certain clinical diagnosis in the presence of two major criteria or one major and two minor criteria; probable FMF—clinical diagnosis considered probable in the presence of one major and one minor criterion or two minor criteria; and non-FMF—clinical diagnosis considered unlikely in the presence of only one minor and no major criteria. Clinical manifestations of FMF, including characteristics of febrile episodes (duration and frequency), and the presence of serositis (chest or abdominal pain), arthritis, myalgia, and erysipelas-like rash was documented. Demographic data (including gender, consanguinity of parents, familial history, and age of onset of inflammation signs) and main clinical data (including fever, thoracic, abdominal, articular, cutaneous signs, duration and frequency of episodes, presence of amyloidosis, and response to colchicine) were recorded by the doctor using a standard form. Response to colchicine was defined as complete, incomplete, or absent.

### Mutational analysis

Blood samples (2 ml) were collected from all subjects. Genomic DNA was extracted from whole blood using the Wizard® Genomic DNA Purification Kit (Promega, USA). Mutational analysis was performed by direct DNA sequencing. Polymerase chain reaction (PCR) amplification was performed for each *MEFV* exon, as described previously [9]. A total of 27 PCR products per patient were purified using ExoSAP-IT (GE Healthcare Japan, Tokyo, Japan) and sequenced directly using specific primers and BigDye Terminator v1.1 (Applied Biosystems, Tokyo, Japan). The control group for *MEFV* genotyping consisted of 105 gender-matched Japanese healthy subjects (44 men and 61 women). The mean ± SD age was 44.2 ± 11.5 years.

### Statistical analyses

Data were analyzed using SPSS software (SPSS Inc., Chicago, IL, USA). Results were expressed as the mean ± standard deviation (SD) for continuous variables. For quantitative data, the Mann–Whitney U rank-sum test compared two independent groups. Comparisons for categorical variables were evaluated using the chi-square test (or Fisher’s exact test when appropriate). Adjustment for multiple comparisons was performed using the Bonferroni method. *Pc* values were calculated by multiplying the *p* value by the number of alleles tested.

## Results

### Patient demographic data

**Fig. 1:**
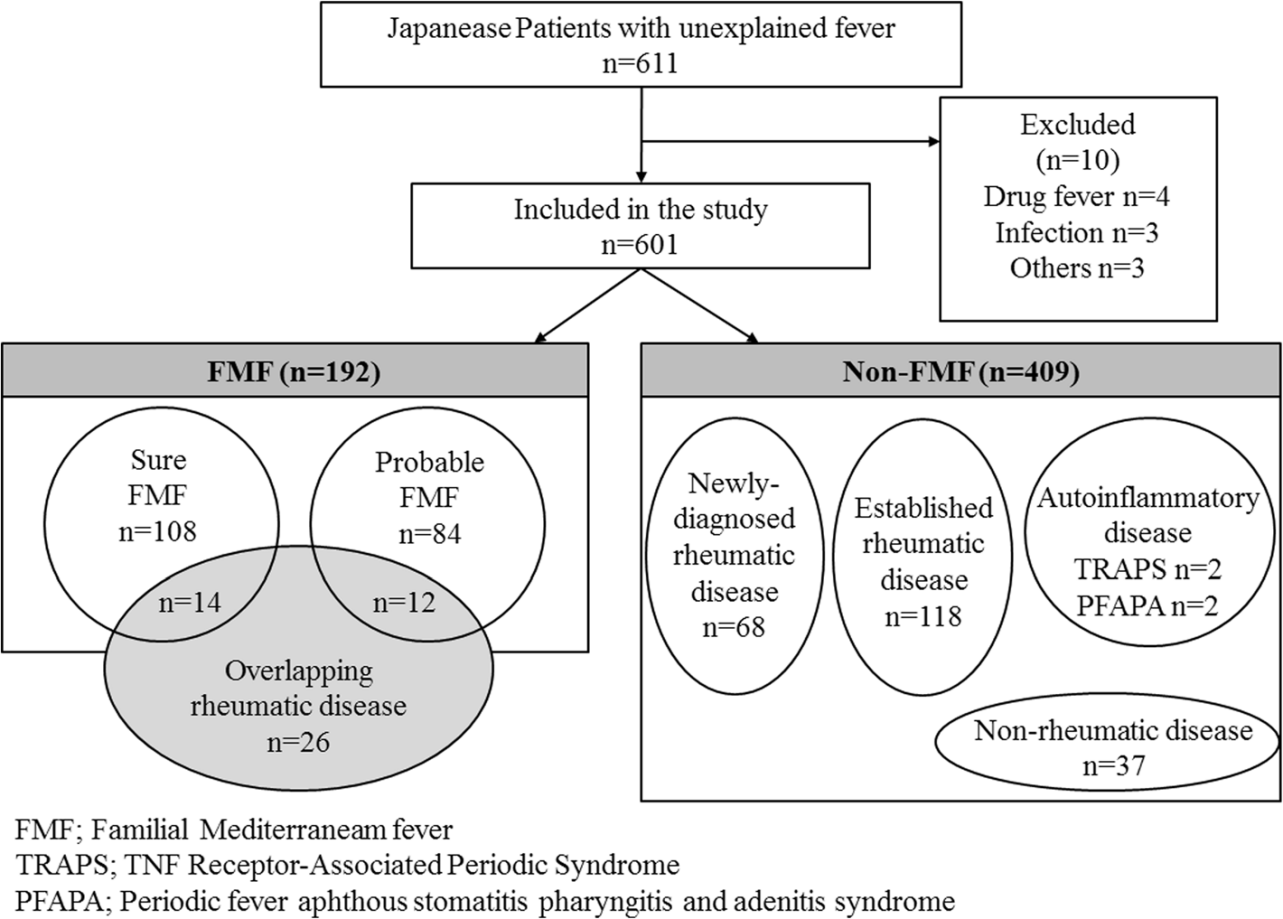
Flow diagram of patient enrollment

Ten patients were excluded from the study (Fig. 1). The main reasons for exclusion were the absence of periodic fever syndrome (drug fever, infections, and neoplastic diseases). At the time of analysis, the mean patient age was 44.3 ± 20.2 years (range 0–94 years) and the mean age of the onset of symptoms was 36.3 ± 19.7 years (range 1–94 years). The main clinical characteristics of the 601 patients were as follows: 385 patients were female (64.1 %), fever was observed in 482 (80.2 %), abdominal symptoms in 163 (27.1 %), thoracic signs in 75 (12.5 %), arthritis signs in 345 (57.4 %), and amyloidosis in 22 (3.7 %). On the basis of Tel Hashomer criteria, 192 patients (31.9 %) were diagnosed with FMF, of whom 108 had typical FMF (56.3 %) and 84 had incomplete FMF (43.7 %). The remaining 409 patients (68.1 %) were classified as non-FMF patients, including two patients with suspected tumor necrosis factor receptor associated periodic syndrome (TRAPS) and two patients with periodic fever, aphthous stomatitis, pharyngitis, and adenitis syndrome (PFAPA) (Fig. 1). As shown in Fig. 1, among non-FMF patients, 118 patients had established rheumatic diseases (rheumatoid arthritis, *n* = 35; systemic lupus erythematosus, *n* = 19; Behçet's disease, *n* = 17; gout, *n* = 12; inflammatory myopathies, *n* = 7; mixed connective-tissue disease, *n* = 4; psoriatic arthritis, *n* = 4; remitting seronegative symmetrical synovitis with pitting edema, *n* = 3; Henoch-Schonlein purpura, *n* = 3; vasculitis syndrome, *n* = 3; SAPHO syndrome, *n* = 2; palindromic rheumatism, *n* = 2; Sjögren's syndrome, *n* = 2; Reiter's syndrome, *n* = 1; Crowned dens syndrome, *n* = 1; relapsing polychondritis, *n* = 1; spondylarthritis, *n* = 1; systemic sclerosis, *n* = 1). Additionally, among non-FMF patients, 68 patients were finally diagnosed as having rheumatic diseases (Behçet's disease, *n* = 11; rheumatoid arthritis, *n* = 9; inflammatory myopathies, *n* = 7; vasculitis syndrome, *n* = 7; Sjögren's syndrome, *n* = 6; systemic lupus erythematosus, *n* = 6; palindromic rheumatism, *n* = 5; mixed connective-tissue disease, *n* = 4; gout, *n* = 3; CREST syndrome, *n* = 2; ankylosing spondylitis, *n* = 2; adult onset Still's disease, *n* = 1; Caplan's syndrome, *n* = 1; IgG4-related disease, *n* = 1; SAPHO syndrome, *n* = 1; psoriatic arthritis, *n* = 1; eosinophilic fasciitis, *n* = 1). Among the remaining non-FMF patients, 37 patients were finally diagnosed with non-rheumatic diseases (amyloidosis, *n* = 5; myelodysplastic syndromes, *n* = 4; Castleman's disease, *n* = 4; undifferentiated arthritis, *n* = 3; viral infection, *n* = 3; Sweet's disease, *n* = 2; Kikuchi's disease, *n* = 2; hemophagocytic syndrome, *n* = 2; chronic thyroiditis, *n* = 2; idiopathic thrombocytopenic purpura, *n* = 1; alcoholic hepatitis, *n* = 1; Wilson's disease, *n* = 1; cryoglobulinemia, *n* = 1; Crohn's disease, *n* = 1; recurrent stomatitis, *n* = 1; malignant lymphoma, *n* = 1; reactive lymphadenitis, *n* = 1; non-tuberculous mycobacterial disease, *n* = 1; interstitial nephritis, *n* = 1).

FMF patients had a shorter duration of febrile attack and higher frequencies of abdominal or thoracic symptoms and family history of periodic fever (Table 1). Colchicine was administered to 300 patients (sure FMF, 86.1 %; probable FMF, 95.2 %; non-FMF patients, 31.1 %), and the response was higher in patients with typical FMF (97.8 %) compared with those in the other groups (non-FMF, 64.6 %). The response rates for colchicine treatment were not significantly different between subgroups of FMF classified by *MEFV* mutations (Table 2).

**Table 1:** Comparisons of clinical features of between FMF and non-FMF

	FMF	Non-FMF	
	*n* = 192	*n* = 409	*p*
Male/female	83/109	133/276	0.011
Age at onset (years), mean ± SD	30.4 ± 39.4	39.4 ± 20.3	<0.0001
Fever	184 (95.8 %)	298 (72.9 %)	<0.0001
Frequencies of febrile attack (per month), mean ± SD	1.06 ± 0.92	0.97 ± 1.11	0.020
Duration of fever attack (days), mean ± SD	3.7 ± 4.0	6.7 ± 8.4	0.001
Abdominal pain	77 (40.1 %)	86 (21.0 %)	<0.0001
Thoracic pain	51 (26.6 %)	24 (5.9 %)	<0.0001
Arthritis	108 (56.3 %)	237 (57.9 %)	0.695
Erysipelas-like erythema	34 (17.7 %)	43 (10.5 %)	0.014
AA amyloidosis	7 (3.6 %)	15 (3.7 %)	0.989
Family history of periodic fever	39 (20.3 %)	25 (6.1 %)	<0.0001
Rheumatic diseases	26 (13.5 %)	186 (45.5 %)	<0.0001

Values are shown as *n* (%) unless otherwise indicated. *AA* amyloid A, *FMF* familial Mediterranean fever

**Table 2:** Clinical response to colchicine in FMF patients

Subgroups			Response rate		*p*
Sure	vs	Probable	91/93 (97.8 %)	74/80 (92.5 %)	0.095
(0.82 ± 0.40)*		(0.84 ± 0.54)*			
*MEFV* mutations (+)	vs	*MEFV* mutations (–)	154/162 (95.1 %)	11/11 (100 %)	0.585
M694I (+)	vs	M694I (–)	36/37 (97.3 %)	129/136 (94.9 %)	0.459
E148Q (+)	vs	E148Q (–)	109/115 (94.8 %)	56/58 (96.6 %)	0.461
Rheumatic diseases (+)	vs	Rheumatic diseases (–)	23/23 (100 %)	142/150 (94.7 %)	0.311

*Mean dose of colchicine, mg/day. *FMF* familial Mediterranean fever

### Clinical manifestations in FMF patients

As shown in Table 3, short durations of fever, and thoracic and abdominal symptoms were more frequently observed in sure FMF patients (2.2 ± 0.8 days) compared with probable FMF patients (6.2 ± 5.5 days). Conversely, arthritis was more frequently observed in probable FMF patients compared with sure FMF patients. Among FMF patients, 7 (3.6 %) had biopsy-proven amyloid A (AA) amyloidosis (sure FMF, *n* = 6; probable FMF, *n* = 1). Among non-FMF patients, 15 patients had AA amyloidosis and primary diseases were rheumatoid arthritis (*n* = 10) and Crohn's disease (*n* = 1), whereas primary diseases were not identified in four patients. The allele frequencies of *MEFV* mutations between AA amyloidosis patients with or without FMF are shown in Table 4. Only the allelic frequency of M694I was significantly higher in FMF patients with AA amyloidosis.

**Table 3:** Comparisons of clinical features of patients with different FMF phenotypes

	Sure FMF	Probable FMF	
	*n* = 108	*n* = 84	*p*
Male/female	51/57	32/52	0.205
Age at onset (years), mean ± SD	30.5 ± 17.4	30.3 ± 16.6	0.939
Fever	108 (100 %)	76 (90.5 %)	0.001
Frequencies of febrile attack (per month), mean ± SD	1.11 ± 0.93	0.98 ± 0.90	0.309
Duration of fever attack (days), mean ± SD	2.2 ± 0.8	6.2 ± 5.5	<0.0001
Abdominal pain	54 (50.0 %)	23 (27.4 %)	0.002
Thoracic pain	33 (30.6 %)	18 (21.4 %)	0.155
Arthritis	53 (49.1 %)	55 (65.5 %)	0.023
Erysipelas-like erythema	15 (13.9 %)	19 (22.6 %)	0.116
AA amyloidosis	6 (5.6 %)	1 (1.2 %)	0.110
Family history of periodic fever	26 (24.1 %)	13 (15.5 %)	0.142
Rheumatic diseases	14 (13.0 %)	12 (14.3 %)	0.790

Values are shown as *n* (%) unless otherwise indicated. *AA* amyloid A, *FMF* familial Mediterranean fever

**Table 4:** Allelic frequencies of *MEFV* mutations of AA amyloidosis patients with or without FMF

		AA amyloidosis		
		FMF	Non-FMF	
		2*n* = 14	2*n* = 30	*p*
Exon10	M694I	8 (57.1 %)	0	<0.0001
Exon3	R408Q	1 (7.1 %)	0	0.318
	P369S	1 (7.1 %)	0	0.318
Exon2	G304R	0	1 (3.3 %)	0.682
	R202Q	1 (7.1 %)	0	0.318
	E148Q	5 (35.7 %)	5 (16.7 %)	0.1540
	L110P	3 (21.4 %)	2 (6.7 %)	0.175
Exon1	E84K	0	1 (3.3 %)	0.682

Values are shown as *n* (%). Primary diseases of amyloid A (AA) amyloidosis in non-familial Mediterranean fever (FMF) were rheumatoid arthritis (*n* = 10) and Crohn's disease (*n* = 1)

For the sure FMF patients, 13.0 % (14/108) had concomitant rheumatic diseases (rheumatoid arthritis, *n* = 6; Sjögren's syndrome, *n* = 3; dermatomyositis complex, *n* = 2; Behçet's disease, *n* = 1; adult onset Still's disease, *n* = 1; Kawasaki disease, *n* = 1). In the probable FMF patients, 14.3 % (12/84) had concomitant rheumatic diseases (systemic lupus erythematosus, *n* = 4; Sjögren's syndrome, *n* = 3; rheumatoid arthritis, *n* = 2; Behçet's disease, *n* = 1; palindromic rheumatism, *n* = 1; polymyositis, *n* = 1).

FMF patients with rheumatic diseases had a higher frequency of arthritis episodes and an elderly onset of FMF. Conversely, FMF patients without rheumatic diseases had a higher frequency of abdominal pain and family history of FMF (Table 5). No significant difference was observed in the allele frequencies in *MEFV* mutations between FMF patients with or without rheumatic diseases (Table 6).

**Table 5:** Comparisons of clinical features of patients with or without accompanying rheumatic diseases

	FMF		
	Rheumatic diseases (+)	Rheumatic diseases (–)	
	*n* = 26	*n* = 166	*p*
Male/female	4/22	79/87	0.002
Age at onset (years), mean ± SD	39.3 ± 15.8	29.0 ± 16.8	0.006
Fever	24 (92.3 %)	160 (96.4 %)	0.296
Frequencies of febrile attack (per month), mean ± SD	1.01 ± 0.59	1.06 ± 0.96	0.523
Duration of fever attack (days), mean ± SD	3.0 ± 2.2	3.8 ± 4.1	0.359
Abdominal pain	3 (11.5 %)	74 (44.6 %)	0.001
Thoracic pain	4 (15.4 %)	47 (28.3 %)	0.165
Arthritis	21 (80.8 %)	87 (52.4 %)	0.007
Erysipelas-like erythema	3 (11.5 %)	31 (18.7 %)	0.281
AA amyloidosis	0	7 (4.2 %)	0.355
Family history of periodic fever	1 (3.8 %)	38 (22.9 %)	0.025

Values are shown as *n* (%) unless otherwise indicated. *AA* amyloid A, *FMF* familial Mediterranean fever

**Table 6:** Comparisons of allelic frequencies of *MEFV* mutations of FMF patients with or without accompanying rheumatic diseases

		FMF			
		Rheumatic diseases (+)	Rheumatic diseases (–)		
		2*n* = 52	2*n* = 332	*p*	*pc*
Exon10	M694I	4 (7.7 %) [1/26 (3.8 %)]	48 (14.5 %) [4/166 (2.4 %)]	0.185	2.7738
	P751L	0	1 (0.3 %)	0.865	12.9687
	G632S	0	1 (0.3 %)	0.865	12.9687
Exon3	R410H	0	1 (0.3 %)	0.865	12.9687
	R408Q	8 (15.4 %) [2/26 (7.7 %)]	23 (6.9 %) [0/166 (0 %)]	0.043	0.6419
	P369S	8 (15.4 %) [2/26 (7.7 %)]	25 (7.5 %) [0/166 (0 %)]	0.060	0.9050
	R354Q	0	1 (0.3 %)	0.865	12.9687
Exon2	G304R	3 (5.8 %) [0/26 (0 %)]	8 (2.4 %) [1/166 (0.6 %)]	0.176	2.6382
	E225K	0	1 (0.3 %)	0.865	12.9687
	R202Q	3 (5.8 %)	14 (4.2 %)	0.411	6.1680
	E148Q	23 (44.2 %) [4/26 (15.4 %)]	127 (38.3 %) [19/166 (11.4 %)]	0.411	6.1702
	P115R	0	0		
	L110P	8 (15.4 %) [0/26 (0 %)]	37 (11.1 %) [1/166 (0.6 %)]	0.377	5.6513
Exon1	E84K	1 (1.9 %)	11 (3.3 %)	0.500	7.4946
	R80H	0	0		

Values are shown as *n* (%) [% of homozygote]. *FMF* familial Mediterranean fever, *pc* corrected *p* value

### Allele frequencies of MEFV mutations in FMF and healthy subjects

Distributions of *MEFV* genotypes in the FMF and non-FMF groups are shown in Table 7. Table 8 shows the allelic frequencies of *MEFV* mutations in the FMF and non-FMF groups. Most FMF patients (94.3 %, 181/192) carried at least one *MEFV* mutation. Significant differences were observed between FMF patients and healthy subjects regarding the allelic frequencies of mutations in *MEFV* exon 2 (E148Q, 39.1 % vs 24.8 %, respectively) and exon 10 (M694I, 13.5 % vs 0 %, respectively). However, there were no significant differences between FMF patients and healthy subjects regarding the allelic frequencies of other mutations (Table 9).

**Table 7:** *MEFV* genotypes in FMF or non-FMF patients

	FMF		Non-FMF		
	Sure	Probable	Newly-diagnosed rheumatic diseases	Established rheumatic diseases	Others
	*n* = 108	*n* = 84	*n* = 68	*n* = 118	*n* = 223
M694I/M694I	5 (4.6 %)	0	0	0	0
M694I/P751L	1 (0.9 %)	0	0	0	0
M694I/E148Q/E148Q	1 (0.9 %)	0	0	0	0
M694I/L110P/E148Q	8 (7.4 %)	0	0	0	0
M694I/E148Q	22 (20.4 %)	0	0	0	0
M694I/normal	10 (9.3 %)	0	0	0	0
G632/E148Q	0	1 (1.2 %)	0	0	0
R354Q/normal	0	1 (1.2 %)	0	0	0
P369S/R408Q	2 (1.9 %)	8 (9.5 %)	2 (2.9 %)	2 (1.7 %)	16 (7.2 %)
G304R/G304R	0	1 (1.2 %)	0	0	0
G304R/P369S/R408Q	0	0	0	0	1 (0.5 %)
G304R/normal	1 (0.9 %)	5 (6.0 %)	1 (1.5 %)	6 (5.1 %)	8 (3.6 %)
R202Q/R202Q	0	0	0	0	1 (0.5 %)
R202Q/P369S/R408Q	1 (0.9 %)	0	0	2 (1.7 %)	0
R202Q/normal	5 (4.6 %)	4 (4.8 %)	4 (5.9 %)	3 (2.5 %)	11 (4.9 %)
E225K/P369S/R408Q	0	1 (1.2 %)	0	0	0
E148Q/E148Q	3 (2.8 %)	4 (4.8 %)	2 (2.9 %)	4 (3.4 %)	2 (0.9 %)
E148Q/G304R/P369S/R408Q	1 (0.9 %)	0	1 (1.5 %)	0	0
E148Q/R202Q/P369S/R408Q	0	2 (2.4 %)	0	0	0
E148Q/R202Q	2 (1.9 %)	0	0	1 (0.9 %)	2 (0.9 %)
E148Q/E148Q/P369S/P369S/R408Q/R408Q	0	1 (1.2 %)	0	0	0
E148Q/E148Q/P369S/R408Q	1 (0.9 %)	2 (2.4 %)	0	0	2 (0.9 %)
E148Q/P369S/P369S/R408Q/R408Q	0	1 (1.2 %)	0	0	1 (0.5 %)
E148Q/P369S/R408Q	3 (2.8 %)	5 (6.0 %)	0	3 (2.5 %)	7 (3.1 %)
E148Q/P369S	0	1 (1.2 %)	0	0	1 (0.5 %)
E148Q/normal	15 (13.9 %)	16 (19.0 %)	10 (14.7 %)	26 (22.0 %)	44 (19.7 %)
P115R/normal	0	0	1 (1.5 %)	1 (0.9 %)	0
L110P/E148Q/E148Q/P369S/R408Q	0	1 (1.2 %)	0	1 (0.9 %)	1 (0.5 %)
L110P/E148Q/E148Q/P369S	0	0	0	1 (0.9 %)	0
L110P/E148Q/R202Q/P369S/R408Q	0	0	0	0	1 (0.5 %)
L110P/E148Q/P369S/R408Q	0	0	1 (1.5 %)	1 (0.9 %)	2 (0.9 %)
L110P/E148Q/P369S	0	1 (1.2 %)	0	0	3 (1.4 %)
L110P/E148Q/G304R	0	1 (1.2 %)	0	0	1 (0.5 %)
L110P/E148Q/R202Q	1 (0.9 %)	2 (2.4 %)	0	0	0
L110P/L110P/E148Q/E148Q	0	0	0	0	1 (0.5 %)
L110P/E148Q/E148Q	3 (2.8 %)	7 (8.3 %)	1 (1.5 %)	1 (0.9 %)	5 (2.2 %)
L110P/L110P/E148Q	1 (0.9 %)	0	0	0	0
L110P/E148Q	13 (12.0 %)	5 (6.0 %)	6 (8.8 %)	13 (11.0 %)	21 (9.4 %)
E84K/L110P/E148Q	0	1 (1.2 %)	0	1 (0.9 %)	0
E84K/G304R	0	1 (1.2 %)	0	0	0
E84K/E148Q	0	2 (2.4 %)	0	0	1 (0.5 %)
E84K/R410H	1 (0.9 %)	0	0	0	1 (0.5 %)
E84K/normal	3 (2.8 %)	4 (4.8 %)	1 (1.5 %)	1 (0.9 %)	6 (2.7 %)
R80H/normal	0	0	0	0	1 (0.5 %)
Normal	5 (4.6 %)	6 (7.1 %)	38 (55.9 %)	51 (43.2 %)	83 (37.2 %)

Values are shown as *n* (%). *FMF* familial Mediterranean fever

**Table 8:** Allele frequencies of *MEFV* mutations in FMF and non-FMF patients

		FMF		Non-FMF			
		Sure	Probable	Newly-diagnosed rheumatic diseases	Established rheumatic diseases	Others	Healthy subjects
		2*n* = 216	2*n* = 168	2*n* = 136	2*n* = 236	2*n* = 446	2*n* = 210
Exon10	M694I	52 (24.1 %)	0	0	0	0	0
	P751L	1 (0.5 %)	0	0	0	0	0
	G632S	0	1 (0.6 %)	0	0	0	0
Exon3	R410H	1 (0.5 %)	0	0	0	1 (0.2 %)	0
	R408Q	8 (3.7 %)	23 (13.7 %)	4 (2.9 %)	9 (3.8 %)	32 (7.2 %)	12 (5.7 %)
	P369S	8 (3.7 %)	25 (14.9 %)	4 (2.9 %)	10 (4.2 %)	36 (8.1 %)	13 (6.2 %)
	R354Q	0	1 (0.6 %)	0	0	0	0
Exon2	G304R	2 (0.9 %)	9 (5.4 %)	3 (1.8 %)	6 (2.4 %)	10 (2.2 %)	6 (2.9 %)
	E225K	0	1 (0.6 %)	0	0	0	0
	R202Q	9 (4.2 %)	8 (4.8 %)	4 (2.9 %)	6 (2.5 %)	16 (3.6 %)	6 (2.9 %)
	E148Q	82 (38.0 %)	68 (40.5 %)	24 (17.6 %)	59 (25.0 %)	106 (23.8 %)	52 (24.8 %)
	P115R	0	0	1 (0.7 %)	1 (0.4 %)	0	0
	L110P	27 (12.5 %)	18 (10.7 %)	8 (5.9 %)	18 (7.6 %)	36 (8.1 %)	15 (7.1 %)
Exon1	E84K	4 (1.9 %)	8 (4.8 %)	1 (0.7 %)	2 (0.8 %)	8 (1.8 %)	2 (1.0 %)
	R80H	0	0	0	0	1 (0.2 %)	0

Values are shown as *n* (%). *FMF* familial Mediterranean fever

**Table 9:** Comparisons of allelic frequencies of *MEFV* mutations between FMF and healthy subjects

		FMF	Healthy subjects		
		2*n* = 384	2*n* = 210	*p*	*pc*
Exon10	M694I	52 (13.5 %)	0	<0.0001	<0.0001
	P751L	1 (0.3 %)	0	0.646	9.6970
	G632S	1 (0.3 %)	0	0.646	9.6970
Exon3	R410H	1 (0.3 %)	0	0.646	9.6970
	R408Q	31 (8.1 %)	12 (5.7 %)	0.289	4.3336
	P369S	33 (8.6 %)	13 (6.2 %)	0.295	4.4222
	R354Q	1 (0.3 %)	0	0.646	9.6970
Exon2	G304R	11 (2.9 %)	6 (2.9 %)	0.996	14.9378
	E225K	1 (0.3 %)	0	0.646	9.6970
	R202Q	17 (4.4 %)	6 (2.9 %)	0.343	5.1459
	E148Q	150 (39.1 %)	52 (24.8 %)	0.0004	0.0065
	P115R	0	0		
	L110P	45 (11.7 %)	15 (7.1 %)	0.077	1.1527
Exon1	E84K	12 (3.1 %)	2 (1.0 %)	0.077	1.1610
	R80H	0	0		

Values are shown as *n* (%). *FMF* familial Mediterranean fever, *pc* corrected *p* value

### MEFV mutations in FMF patients

Table 10 shows the allelic frequencies of *MEFV* mutations according to the FMF disease phenotype. Among FMF patients, the allelic frequencies of the *MEFV* exon 10 mutation (M694I) were significantly higher in sure FMF patients compared with probable FMF patients, while the allelic frequencies of the *MEFV* exon 3 mutations (P369S, R408Q) were significantly lower in sure FMF patients compared with probable FMF patients. No significant difference was seen in the allele frequencies of other *MEFV* mutations between FMF patients with or without rheumatic disease.

**Table 10:** Comparisons of allelic frequencies of *MEFV* mutations of patients with sure FMF and probable FMF

		Sure FMF	Probable FMF		
		2*n* = 216	2*n* = 168	*p*	*pc*
Exon10	M694I	52 (24.1 %)	0	<0.0001	<0.0001
	P751L	1 (0.5 %)	0	0.563	8.4375
	G632S	0	1 (0.6 %)	0.438	6.5625
Exon3	R410H	1 (0.5 %)	0	0.563	8.4375
	R408Q	8 (3.7 %)	23 (13.7 %)	0.0004	0.0055
	P369S	8 (3.7 %)	25 (14.9 %)	0.0001	0.0016
	R354Q	0	1 (0.6 %)	0.438	6.5625
Exon2	G304R	2 (0.9 %)	9 (5.4 %)	0.011	0.1641
	E225K	0	1 (0.6 %)	0.438	6.5625
	R202Q	9 (4.2 %)	8 (4.8 %)	0.778	11.6771
	E148Q	82 (38.0 %)	68 (40.5 %)	0.617	9.2482
	P115R	0	0		
	L110P	27 (12.5 %)	18 (10.7 %)	0.589	8.8411
Exon1	E84K	4 (1.9 %)	8 (4.8 %)	0.104	1.5597
	R80H	0	0		

Values are shown as *n* (%). *FMF* familial Mediterranean fever, *pc* corrected *p* value

### Influence of MEFV mutation number on clinical phenotype

Although FMF is considered an autosomal recessive disease, the presence of only a single mutation can often be associated with the occurrence of FMF. We analyzed the differences in clinical manifestations according to the number of *MEFV* mutations. FMF patients with two or more than two *MEFV* mutations had AA amyloidosis and family history of periodic fever more frequently compared with those with a single or no *MEFV* mutations (Table 11).

**Table 11:** Clinical features of FMF patients with different numbers of *MEFV* mutations

	No. of *MEFV* mutations		
Clinical manifestations	≥2	0 or 1	
	*n* = 117	*n* = 75	*p*
Male/female	52/65	31/44	0.671
Age at onset (years), mean ± SD	28.3 ± 15.2	33.7 ± 19.2	0.097
Fever	113 (96.6 %)	71 (94.7 %)	0.383
Frequencies of febrile attack (per month), mean ± SD	1.11 ± 0.97	0.97 ± 0.83	0.438
Duration of fever attack (days), mean ± SD	3.1 ± 2.1	4.6 ± 5.6	0.201
Abdominal pain	51 (43.6 %)	26 (34.7 %)	0.218
Thoracic pain	36 (30.8 %)	15 (20.0 %)	0.099
Arthritis	69 (59.0 %)	39 (52.0 %)	0.342
Erysipelas-like erythema	17 (14.5 %)	17 (22.7 %)	0.150
AA amyloidosis	7 (6.0 %)	0	0.029
Family history of periodic fever	31 (26.5 %)	8 (10.7 %)	0.008
Rheumatic diseases	18 (15.4 %)	8 (10.7 %)	0.351

Values are shown as *n* (%) unless otherwise indicated. *AA* amyloid A, *FMF* familial Mediterranean fever

## Discussion

This is a multicentric study into the prevalence of FMF patients in Japan. FMF was diagnosed in a high number of Japanese patients with unexplained fever or rheumatic manifestations. Based on our findings, we propose that FMF should be considered as a differential diagnosis for patients with unexplained rheumatic symptoms, even in the Japanese population. Other forms of recurrent hereditary fever, such as TRAPS, appear to be rarer than FMF, although genetic analysis for these diseases was not routinely performed.

The clinical diagnosis of FMF is not easy [3]. It has mainly been based on clinical signs, although *MEFV* genetic analysis is useful in Japan [14]. It is conceivable that the reported delay in diagnosis may result from the low awareness of FMF in Japan because of its misconceived rarity. The detection of *MEFV* mutations with high penetrance may help achieve a precise FMF diagnosis [15]. However, the observation of many heterozygous patients in whom a second allele was excluded [7, 16], especially in non-Mediterranean countries such as Japan, suggests the involvement of other genetic or environmental FMF susceptibility factors in disease susceptibility [17, 18]. Additionally, *MEFV* variants with low penetrance could be associated with clinical features that resemble FMF [10, 11].

It is evident that the use of the genetic approach to FMF diagnosis in patients with atypical clinical presentations has not been fully addressed. Of note, we identified significant differences in the allele frequencies of *MEFV* variants (M694I and E148Q) between FMF and non-FMF patients in the present study. The diagnostic value of *MEFV* exon 10 mutations has previously been established [19]; however, *MEFV* exon 2 or 3 polymorphisms were not thought to affect FMF occurrence [20, 21]. The E148Q variant has been established as a polymorphism, but some studies suggest that it is related to some clinical manifestations of rheumatic diseases [22]. In our study, the prevalence of this *MEFV* variant was increased in FMF patients compared with health subjects.

Our Japanese FMF patients had some notable clinical and genetic characteristics. The prevalence of *MEFV* exon 10 mutations and *MEFV* homozygous mutations was lower compared with those in Western countries [23]. Contrary to the concept that FMF is caused by recessive loss-of-function mutations, it is more likely that *MEFV* mutations cause FMF by a gain-of-function model [24]. It is conceivable that these genetic features contribute to the increased proportion of patients with probable FMF. Additionally, a genotype–phenotype relationship between the *MEFV* exon 10 mutation and the sure FMF phenotype was confirmed. Although clinical judgments still remain crucial in FMF diagnosis, our data show that a molecular approach to FMF diagnosis enables confirmation of typical FMF cases or genotype–phenotype correlations.

In the present study, we defined a minor subgroup carrying *MEFV* variants in whom a definitive diagnosis of FMF was made in addition to pre-existing established rheumatic diseases. These patients had periodic fever, serositis, or synovitis that was not explained by the activities of primary rheumatic diseases. Furthermore, these clinical manifestations were silenced by colchicine in the majority of patients. These findings suggest that an overlap between FMF and established rheumatic diseases is not unusual. It is well known that rheumatic diseases including lupus often cause acute serositis. When FMF patients with these rheumatic diseases showed laboratory data suggestive of active primary rheumatic diseases, such as hypocomplementemia or high titer of anti-ds-DNA antibody, these manifestations seem to be caused by FMF-related serositis [25]. Additionally, steroids have no beneficial effects in FMF attacks. A response to adequate colchicine therapy could confirm FMF [26], whereas steroid use has a benefit in some autoinflammatory diseases, including AOSD [27]. These findings may provide valuable information on differential diagnosis for FMF and rheumatic diseases.

Conflicting evidence exists as to whether single *MEFV* mutations are associated with the occurrence of other inflammatory diseases [28]. *MEFV* has previously been shown to be an independent modifier of the clinical manifestations of rheumatoid arthritis. Rabinovich et al. found that rheumatoid arthritis patients carrying *MEFV* mutations developed more severe disease than those with multiple mutations [22], while Ayaz et al. reported that juvenile idiopathic arthritis patients harboring *MEFV* mutations presented with the polyarticular course with detective arthritis [29]. These findings suggest that *MEFV* mutations or polymorphisms, even in one allele, associate with atypical clinical manifestations or subclinical inflammation not attributable to the primary rheumatic disease. It is tempting to speculate that, after the development of rheumatic diseases, the presence of an *MEFV* mutation modulates the clinical phenotype or contributes to the occurrence of FMF. No consensus has yet been demonstrated to classify the E148Q variant as pathogenic or non-pathogenic [30]. This sequence variant was described as a disease-causing mutation with low penetrance [31]. On the other hand, 50 % of E148Q homozygotes are asymptomatic and there is high prevalence of this variant in the Japanese population contrasting with a low FMF prevalence. E148Q is insufficient to trigger FMF but may act as a disease modifier [32]. In our study, Japanese FMF patients have a higher prevalence of E148Q compared to healthy subjects. Although the allele frequency of the E148Q variant is high in the Japanese population, these data may suggest that some Japanese patients with low-penetrance E148Q mutation may develop FMF in combination with unknown environmental or other genetic factors.

Our present study has a number of limitations. One of the main limitations of our study may be its hospital-based nature. The prevalence of symptomatic or FMF-suspicious individuals may be higher in those patients attending hospital regularly. Also, we did not evaluate disease severity, and there was insufficient follow-up of the long-term disease course, including the response to colchicine treatment. Although participating hospitals were encouraged to update patient files, these measures are not complete. The regular screening for AA amyloidosis was not performed in some institutes, which may alter the incidence of AA amyloidosis in our subjects. The mean age of onset of FMF patients in this study was 28.4 years, which seems to be relatively older compared to the previous Japanese investigations. A significant number of enrolments of adult patients with FMF may contribute to the more elderly onset of FMF in this study.

## Conclusions

Our data showed a high prevalence of FMF as well as *MEFV* mutations in Japanese patients with unexplained fever. We suggest that a significant number of FMF cases were included in Japanese patients with unexplained fever. Mutational analysis of *MEFV* should be considered in cases of unexplained fever or non-specific rheumatic manifestations, even in Japan.

## Abbreviations

AA, amyloid A; FMF, familial Mediterranean fever; *MEFV*, MEditerranean FeVer gene; SAPHO, Synovitis Acne Pustulosis Hyperostosis Osteitis
